# Simvastatin as a Biological Adjunct to Guided Bone Regeneration and Related Implant Regenerative Procedures: A Systematic Review

**DOI:** 10.7759/cureus.105665

**Published:** 2026-03-22

**Authors:** Shubham B Tale, Rajesh Gaikwad, Akshaya Banodkar, Harshda S Mahajan, Pranav Kudale, Nandini Metaliya, Sanpreet S Sachdev

**Affiliations:** 1 Department of Periodontology, Government Dental College and Hospital, Mumbai, IND; 2 Department of Oral Pathology and Microbiology, Bharati Vidyapeeth (Deemed to be University) Dental College and Hospital, Navi Mumbai, IND

**Keywords:** bone regeneration, dental implants, guided bone regeneration, immediate implant placement, simvastatin

## Abstract

Tooth extraction is followed by progressive alveolar ridge resorption, which can compromise implant positioning, peri-implant hard tissue support, and esthetic outcomes. Guided bone regeneration (GBR) and related implant-site regenerative approaches are therefore frequently used to improve peri-implant healing. Simvastatin, a 3-hydroxy-3-methylglutaryl-coenzyme A (HMG-CoA) reductase inhibitor, has shown osteopromotive, anti-inflammatory, and angiogenic effects and has recently been explored as a local adjunct in implant-associated regenerative procedures. This systematic review evaluated the available human and animal evidence on localized simvastatin used with GBR or related regenerative protocols during implant placement.

This systematic review was conducted according to Preferred Reporting Items for Systematic Reviews and Meta-Analyses (PRISMA) 2020 guidelines and registered in PROSPERO (CRD420251037455). Electronic searches of PubMed/MEDLINE, Embase, Scopus, Web of Science, Cochrane CENTRAL, Google Scholar, and ClinicalTrials.gov were performed from database inception to April 30, 2025. Randomized controlled trials (RCTs), controlled clinical studies, and animal studies evaluating local simvastatin in implant-associated regenerative procedures were eligible. Data were synthesized qualitatively because of heterogeneity in study design, simvastatin delivery systems, comparator protocols, and outcome measures.

Five studies were included: three human clinical studies and two animal studies. In the human studies, simvastatin was associated with reduced peri-implant bone loss (0.975±0.0438 mm versus 1.356±0.0384 mm; P<0.0001), improved probing depth and bleeding scores, greater bone width gain (5.55±0.80 mm versus 3.36±0.19 mm), and higher bone mineral density (640.02±262.22 versus 297.38±82.94) compared with controls. In the animal studies, simvastatin improved bone-to-implant contact and bone area; for example, grafted bone-to-implant contact at four weeks increased from 59.5% in controls to 84.3% in simvastatin-treated sites, while grafted bone area increased from 51.4% to 75.9%. Implant stability findings were mixed, and no major adverse effects were reported.

The available evidence suggests that locally delivered simvastatin may improve hard tissue regeneration and selected peri-implant clinical outcomes when used with GBR or related implant regenerative protocols. However, the evidence base is limited by the small number of studies, mixed human and animal designs, and heterogeneity in simvastatin concentration, carrier systems, and follow-up periods. Larger, well-designed clinical trials with standardized protocols are needed before routine clinical use can be recommended.

## Introduction and background

Tooth extraction initiates a cascade of biological events that leads to progressive alveolar ridge resorption, producing clinically significant changes in both horizontal and vertical bone dimensions [1,2]. This remodeling process is most pronounced along the buccal aspect of the ridge, where thin cortical plates are particularly prone to collapse following loss of periodontal ligament stimulation [3,4]. Evidence indicates that nearly 50% of ridge width may be lost within the first year after extraction, with approximately two-thirds occurring during the initial three months [5]. Such rapid resorption frequently results in ridge deformities that compromise prosthetically driven implant placement, negatively affecting implant positioning, primary stability, and esthetic outcomes, especially in the anterior maxilla, where cortical plates are inherently thin [6,7]. In this context, preservation and reconstruction of alveolar bone are essential for predictable implant therapy.

Immediate implant placement has gained popularity because it can reduce treatment time and help preserve peri-implant soft tissue contours; however, it does not halt the physiological resorption that follows extraction [8-10]. Anatomically, the diameter mismatch between the implant and the extraction socket leads to horizontal peri-implant gaps that are most evident on the buccal side [11]. Without augmentation, these voids are prone to incomplete mineralization or fibrous tissue replacement, resulting in marginal defects, fenestrations, or compromised facial bone thickness that may threaten esthetics and osseointegration [12]. Consequently, guided bone regeneration (GBR) has become integral to immediate implant protocols, functioning to fill peri-implant defects, preserve ridge dimensions, and support long-term implant stability [13,14].

GBR is founded on the principle of excluding non-osteogenic soft tissue cells while maintaining a secluded space for osteogenic cell proliferation and maturation [15,16]. Barrier membranes, either resorbable collagen-based options or non-resorbable materials such as expanded polytetrafluoroethylene, protect this regenerative environment by providing structural stability and ensuring space maintenance [17-19]. Graft materials placed beneath the membrane act as scaffolds, supporting cellular infiltration and new bone deposition [20,21]. When properly executed, GBR has demonstrated consistent clinical success, enabling horizontal and vertical augmentation, enhancing implant placement accuracy, and improving esthetic outcomes [22,23]. Nonetheless, complications such as membrane exposure, soft tissue dehiscence, infection, and variable graft resorption underscore its technique sensitivity and limitations [24,25].

To address these challenges, biologically active adjuncts have been incorporated into traditional GBR protocols in an effort to enhance osteogenesis, angiogenesis, and overall regenerative capacity [26-28]. Among these, simvastatin has emerged as a promising osteoinductive agent with documented effects on bone metabolism. Beyond its lipid-lowering role as a 3-hydroxy-3-methylglutaryl-coenzyme A (HMG-CoA) reductase inhibitor [29,30], simvastatin stimulates bone morphogenetic protein-2 (BMP-2) expression, promotes osteoblast proliferation and differentiation, suppresses osteoclast activity, and exerts anti-inflammatory and angiogenic effects, all of which contribute synergistically to bone regeneration [31-34]. Preclinical models have demonstrated that locally delivered simvastatin enhances bone formation in extraction sockets and critical-size defects [31,32,35,36], especially when incorporated into controlled-release carriers such as scaffolds, gels, or membranes [37]. Early clinical applications in implant dentistry suggest that simvastatin-augmented implant-related regenerative procedures may improve bone volume gain, bone-to-implant contact, and stability outcomes [38,39]. However, the currently available evidence remains limited, methodologically heterogeneous, and distributed across both human and animal studies, making definitive clinical interpretation difficult.

Given the growing interest in biologically enhanced GBR and the lack of consolidated evidence on immediate implant placement, a systematic evaluation of simvastatin as an adjunctive regenerative agent is warranted. At present, the available literature is sparse; varies in simvastatin concentration, delivery vehicle, grafting approach, membrane use, and outcome assessment; and has not been synthesized specifically in relation to implant-associated regenerative procedures. The present systematic review critically assesses available evidence comparing conventional GBR with simvastatin-supplemented GBR, or closely related implant regenerative protocols using local simvastatin, thereby guiding clinicians toward more predictable, biologically optimized regenerative strategies.

## Review

Methodology

The present systematic review was conducted according to the Preferred Reporting Items for Systematic Reviews and Meta-Analyses (PRISMA) 2020 guidelines [40], and the protocol was registered in the PROSPERO database (reference ID: CRD420251037455). The review was designed to evaluate the available human and animal evidence on the localized use of simvastatin in implant-associated regenerative procedures, particularly in immediate or simultaneous implant placement settings involving guided bone augmentation or related peri-implant regenerative approaches.

Search Strategy

A structured and comprehensive search strategy was implemented to identify eligible studies. Electronic databases, including PubMed/MEDLINE, Embase, Scopus, Web of Science, and Cochrane CENTRAL, were searched from their inception to April 30, 2025. No language restrictions were applied during the electronic search. Grey literature sources, including Google Scholar and ClinicalTrials.gov, were also screened to minimize publication bias. The search strategy incorporated combinations of keywords, controlled vocabulary terms, Boolean operators, and truncations related to guided bone regeneration, simvastatin, dental implants, immediate implant placement, simultaneous implant placement, peri-implant bone regeneration, ridge splitting, osteogenesis, and bone regeneration. The Boolean operator “OR” was used to combine synonyms within each concept, while “AND” was used to combine the major concepts of simvastatin, implant-related regenerative procedures, and bone regeneration. Reference lists of included studies were manually screened to identify additional eligible articles. The complete database-wise search strategies are provided in the Appendices.

Eligibility Criteria

Study selection was guided by the Population, Intervention, Comparison, Outcomes, and Study Design (PICOS) framework to ensure the inclusion of studies addressing the focused clinical question. The population of interest comprised human participants and animal models undergoing implant-associated regenerative procedures, including immediate implant placement, simultaneous implant placement, or peri-implant defect regeneration. In vitro studies were not included in the final eligibility criteria, as the focus of the present review was on in vivo regenerative outcomes with direct translational relevance. The intervention of interest was the localized use of simvastatin as an adjunct to guided bone regeneration or related implant-site regenerative procedures, irrespective of the delivery method, such as gels, scaffolds, coated membranes, or graft-incorporated formulations. Studies using systemic simvastatin or combining simvastatin with other adjuncts without a comparator arm were excluded. The comparison group consisted of conventional guided bone regeneration procedures or comparable regenerative implant protocols without simvastatin. Eligible outcomes included clinical and radiographic parameters such as bone fill, vertical or horizontal bone gain, marginal bone loss, implant stability values, soft tissue healing, bone density, and histological indicators of osseointegration and new bone formation. Only studies providing quantitative assessments were included. Eligible study designs consisted of randomized controlled trials (RCTs), controlled clinical trials, prospective clinical studies, and animal studies, while case reports, case series without comparison groups, reviews, editorials, conference abstracts, and non-comparative experimental reports were excluded. The focused PICOS question was as follows: in implant-associated regenerative procedures, does localized simvastatin used as an adjunct to GBR or related regenerative protocols improve hard and soft tissue outcomes compared with comparable protocols without simvastatin?

Study Selection and Data Extraction

Two independent reviewers (ST and RG) screened the titles and abstracts obtained through the search strategy. Full texts of potentially relevant articles were then evaluated based on the eligibility criteria. Any disagreements in study selection were resolved by discussion with a third reviewer (AB). Reasons for exclusion at the full-text stage were recorded and presented in the PRISMA flow diagram. Data extraction was performed using a standardized spreadsheet, recording details such as author and year of publication, country, study design, sample size, characteristics of the study population or animal model, the form and concentration of simvastatin used, the type of guided bone regeneration or regenerative protocol performed, the comparison protocol, follow-up duration, outcomes measured, quantitative findings, conclusions, funding sources, and declared conflicts of interest. This structured approach ensured uniform data capture and minimized errors during extraction. A qualitative synthesis of the data was performed. In view of the limited number of studies and the substantial heterogeneity in study design, simvastatin delivery systems, comparator protocols, follow-up periods, and reported outcomes, meta-analysis was not considered appropriate. The findings were therefore synthesized narratively and grouped according to study type (clinical versus animal studies) and major outcome domains.

Risk of Bias Assessment

The risk of bias for each study was assessed independently by two reviewers (ST and RG) with disagreements resolved through consensus, and if required, after consultation with a third reviewer (AB). The risk of bias in the randomized controlled trials was evaluated using the Cochrane Risk of Bias-2 tool, which considers potential biases related to randomization, deviations from intended interventions, missing outcome data, measurement of outcomes, and selective reporting [41]. Non-randomized studies were assessed using the ROBINS-I tool, addressing bias due to confounding, participant selection, classification of interventions, deviations from intended protocols, missing data, outcome measurement, and selective reporting [42]. The animal studies were appraised using the SYRCLE tool, which evaluates sequence generation, baseline characteristics, allocation concealment, random housing, blinding of caregivers and investigators, random outcome assessment, and blinding of outcome assessment [43]. The overall risk of bias for each study was determined by synthesizing judgments across domains.

Results

**Figure 1:**
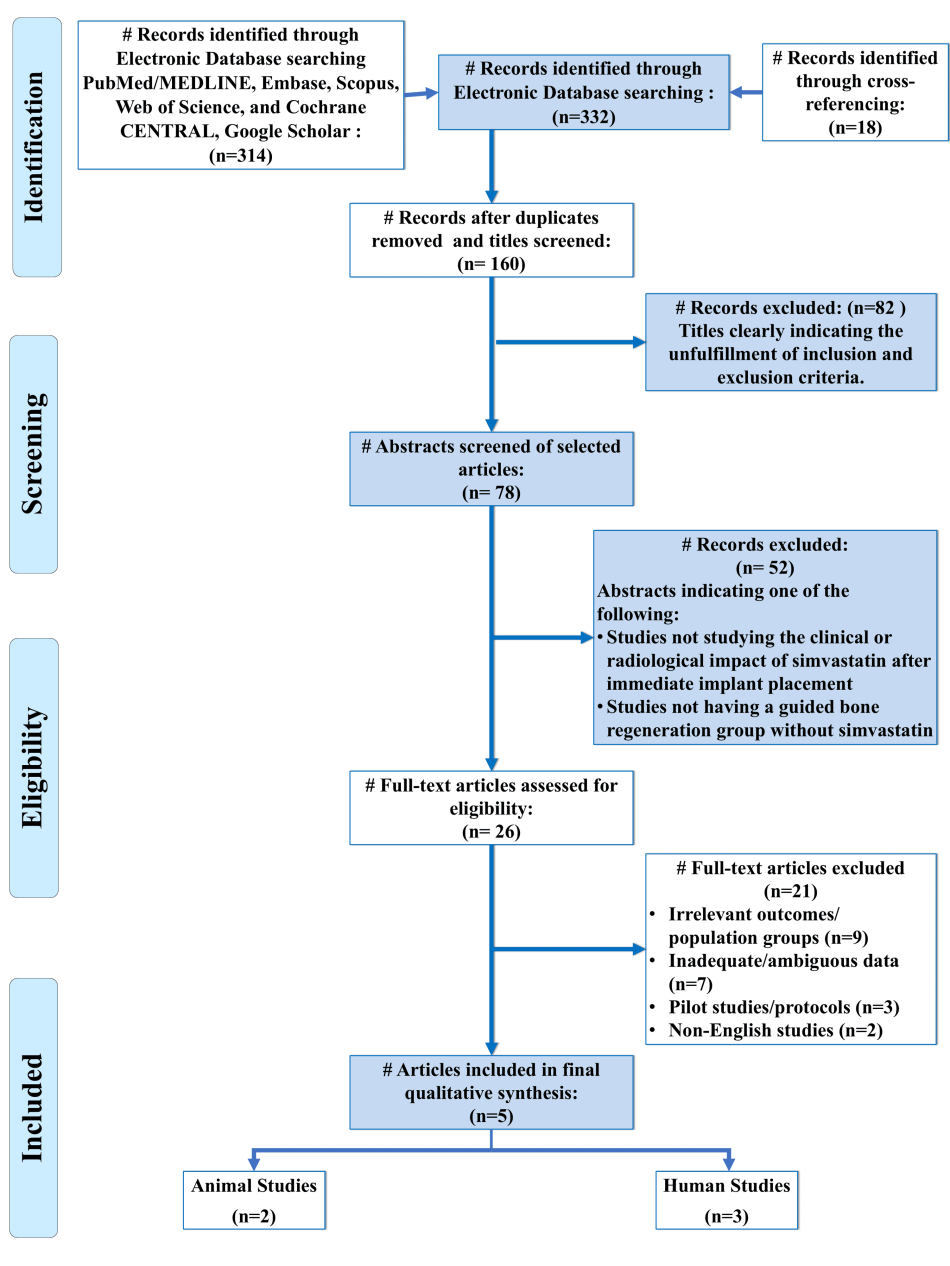
PRISMA flow diagram indicating the selection process of the records in the present systematic review PRISMA: Preferred Reporting Items for Systematic Reviews and Meta-Analyses

A total of five studies met the predefined inclusion criteria and were included in this systematic review (Figure 1) [44-48]. These comprised three human clinical studies and two preclinical animal studies. No in vitro studies met the final eligibility criteria.

The extracted data are presented separately for human clinical studies (Table 1) and preclinical animal studies (Table 2) to improve readability and to distinguish outcomes of direct clinical relevance from mechanistic preclinical findings. The included studies were conducted across three countries, with the majority from Egypt (n=3) and one study each from India and South Korea [44-48]. This distribution reflects a limited but emerging body of evidence on the use of simvastatin in implant-associated regenerative procedures.

**Table 1:** Data extracted from the included human studies related to their characteristics and outcomes SMV: simvastatin, GBR: guided bone regeneration, PRF: platelet-rich fibrin, IIP: immediate implant placement, RCT: randomized controlled trial, CBL: crestal bone level, ISQ: implant stability quotient, PD: probing depth, BOP: bleeding on probing, PI: plaque index, PPD: probing pocket depth, PES: Pink Esthetic Score, MBL: marginal bone level, BMD: bone mineral density, NS: not significant, mo: months, wk: weeks, mg: milligram, mm: millimeter, yrs: years

Author (year)	Country	Study type	Sample size	Population	SMV intervention	Comparator	GBR/graft membrane	Follow-up	Outcomes	Key quantitative results	Key findings
El Shafei et al. (2022) [44]	Egypt	Non-randomized split-mouth clinical trial	8 patients, 16 implants	Male patients, 45-60 yrs, Kennedy class I mandible	PRF scaffold loaded with 1.2 mg SMV applied locally in the osteotomy site during IIP	PRF alone	PRF scaffold; no additional graft/membrane	12 mo	CBL changes, ISQ	Mean bone change (0-12 mo): SMV/PRF 0.975±0.0438 mm versus PRF 1.356±0.0384 mm (P<0.0001); ISQ: NS	SMV/PRF reduced peri-implant bone resorption versus PRF alone; no significant difference in implant stability
Betha et al. (2024) [45]	India	RCT	50 patients	Healthy non-smokers requiring IIP	1% SMV gel applied to the extraction socket before implant placement	IIP without SMV	No graft or membrane	12 wk	PD, BOP, CBL	PD: 5.7±0.9 to 3.2±0.6 mm with SMV versus 5.8±1.1 to 4.1±0.8 mm without SMV; BOP: 1.4±0.5 to 0.6±0.3 versus 1.3±0.4 to 0.9±0.2; CBL: 8.2±1.5 to 8.6±1.2 mm versus 8.3±1.4 to 7.2±1.1 mm	SMV improved crestal bone preservation, reduced inflammation, and enhanced peri-implant clinical parameters
Issa et al. (2024) [48]	Egypt	RCT	22 patients, 49 implants	Adults, 20-55 yrs, horizontally atrophic ridges, non-smokers, medically healthy	1.2% SMV gel mixed with Tutobone and covered with Tutopatch during ridge splitting and simultaneous implant placement	Same surgical protocol with Tutobone + Tutopatch, without SMV	Xenograft + resorbable membrane	6 and 9 mo	PI, BOP, PPD, PES, bone width, MBL, BMD	PES: 11.50±0.91 versus 10.01±0.68 at 6 mo; 13.50±0.51 versus 11.07±0.63 at 9 mo; bone width gain at 9 mo: 5.55±0.80 mm versus 3.36±0.19 mm; MBL loss at 9 mo: 0.96±0.25 mm versus 0.94±0.12 mm; BMD gain at 9 mo: 640.02±262.22 versus 297.38±82.94	SMV with GBR during ridge splitting improved bone density, ridge width, and esthetic outcomes without complications

**Table 2:** Characteristics and outcomes of included preclinical animal studies SMV: simvastatin, GBR: guided bone regeneration, HPMC: hydroxy propyl methyl cellulose, IIP: immediate implant placement, UV: ultraviolet, BIC: bone-to-implant contact, BA: bone area

Author (year)	Country	Study type	Sample size	Population	SMV intervention	Comparator	GBR/graft membrane	Follow-up	Outcomes	Key quantitative results	Key findings
Mansour et al. (2014) [46]	Egypt	Animal study (dog model)	10 dogs	Healthy adult mongrel dogs, 18-24 months	2.2 mg SMV granules in HPMC matrix packed into the socket during IIP	IIP without SMV	SMV granules used as grafting material; no conventional membrane/graft	1 and 3 mo	Histological bone formation, neovascularization, osseointegration, collagen deposition	SMV sites showed greater new bone formation, higher osteocyte count, and implant serration notching; controls showed minimal bone fill and more connective tissue with lower vascularity	Topical SMV enhanced bone regeneration, vascularization, and osseointegration around immediate implants
Jun et al. (2021) [47]	South Korea	Animal study (rabbit model)	12 rabbits, 48 implants	Healthy female New Zealand white rabbits, 3 months old	Implants immersed in 0.5 mM SMV for 24 h, with or without UV treatment	Untreated implants; UV-only implants	Bio-Oss xenograft + GENOSS resorbable collagen membrane in peri-implant defects	2 and 4 wk	BIC, BA	Nongrafted BIC: control 59.3% (2 wk), 66.2% (4 wk) versus SMV 80.9% (2 wk), 85.2% (4 wk); grafted BIC at 4 wk: control 59.5% versus SMV 84.3%; grafted BA at 4 wk: control 51.4% versus SMV 75.9%	SMV-coated implants enhanced BIC and BA with or without grafting; no synergistic benefit with UV+SMV

Study Characteristics

Of the five included studies, three were human clinical studies, including two randomized controlled trials and one non-randomized split-mouth clinical trial, while two were preclinical animal studies involving dog and rabbit models [44-48]. The human studies included 8-50 patients, whereas the animal studies included 10 dogs and 12 rabbits. All studies involved healthy subjects or animal models, with exclusion of smoking or systemic disease in the human trials to reduce confounding factors. Because of this limited and heterogeneous evidence base, the results were synthesized narratively rather than statistically pooled.

The interventions across all studies involved the local delivery of simvastatin, either as a gel, scaffold-incorporated matrix, granules, or implant surface coating. Simvastatin concentrations varied, with commonly used formulations including 1%, 1.2%, and 0.5 mM, depending on the study design. These interventions were applied in regenerative settings that differed in their use of graft materials and membranes. Two studies used xenograft-based regenerative protocols with resorbable membranes, one used PRF as the only scaffold, one used simvastatin granules without a conventional GBR membrane, and one evaluated immediate implant placement without graft or membrane support [44-48]. This variability in regenerative design should be considered when interpreting the findings.

The comparator groups in all studies consisted of subjects or animal sites treated with similar implant-related regenerative protocols without simvastatin, allowing evaluation of the adjunctive effect of local simvastatin. Follow-up durations ranged from two weeks to 12 months, enabling assessment of both early healing responses and longer-term peri-implant changes.

Human Clinical Evidence

The three human studies evaluated a range of clinical and radiographic outcomes, including crestal bone level or marginal bone loss, probing depth, bleeding on probing, implant stability quotient (ISQ), bone width gain, bone mineral density, and esthetic scores [44,45,48]. Overall, simvastatin-containing groups showed more favorable hard and soft tissue outcomes than their respective controls, although the magnitude and consistency of benefit varied between studies.

El Shafei et al. reported that PRF loaded with 1.2 mg simvastatin produced significantly less peri-implant bone loss over 12 months than PRF alone (0.975±0.0438 mm versus 1.356±0.0384 mm; P<0.0001), while implant stability quotient values did not differ significantly between groups [44]. Betha et al. found that 1% simvastatin gel used at the extraction socket during immediate implant placement improved peri-implant clinical parameters, with probing depth reducing from 5.7±0.9 mm to 3.2±0.6 mm in the simvastatin group compared with 5.8±1.1 mm to 4.1±0.8 mm in controls, along with greater reduction in bleeding on probing and better crestal bone preservation [45]. Issa et al. reported that simvastatin mixed with xenograft and covered by a resorbable membrane during ridge splitting and simultaneous implant placement significantly improved esthetic and hard tissue outcomes, including greater bone width gain at nine months (5.55±0.80 mm versus 3.36±0.19 mm) and higher bone mineral density (640.02±262.22 versus 297.38±82.94), while marginal bone loss remained comparable between groups [48].

Taken together, the human data suggest that local simvastatin may contribute to better crestal bone preservation, improved peri-implant soft tissue parameters, greater bone width gain, and higher bone density, although implant stability did not show consistent improvement across studies.

Preclinical Animal Evidence

The two animal studies provided mechanistic and histological support for the regenerative potential of simvastatin in implant-related healing [46,47]. Mansour et al. demonstrated that 2.2 mg simvastatin granules in a hydroxypropyl methylcellulose matrix enhanced new bone formation, osteocyte presence, vascularity, and osseointegration in extraction sockets receiving immediate implants, compared with untreated control sites [46]. Histologically, the simvastatin-treated sites showed more mature bone fill and less intervening connective tissue, supporting a positive biological effect on socket healing and peri-implant regeneration.

Jun et al. reported that simvastatin-coated implants improved bone-to-implant contact and bone area in a rabbit peri-implant defect model, both with and without grafting [47]. In nongrafted sites, bone-to-implant contact increased from 59.3% to 80.9% at two weeks and from 66.2% to 85.2% at four weeks in the simvastatin-treated group. In grafted defects, bone-to-implant contact at four weeks was 84.3% with simvastatin versus 59.5% in controls, while bone area reached 75.9% versus 51.4%, respectively [47]. No synergistic benefit was observed when simvastatin was combined with ultraviolet treatment.

Overall, the animal data consistently support enhanced osseointegration, vascularization, and new bone formation with local simvastatin delivery, thereby providing biological plausibility for the favorable trends observed in the human studies.

Overall Outcome Trends

Across both human and animal evidence, the most consistent benefit of simvastatin was observed in hard tissue regenerative outcomes, including reduced crestal bone loss, increased bone width, improved bone density, higher bone-to-implant contact, and greater bone area [44-48]. Soft tissue and inflammatory parameters also improved in some clinical studies, particularly probing depth, bleeding on probing, and pink esthetic scores [45,48]. However, implant stability as measured by ISQ did not show significant improvement where assessed, suggesting that the biological advantages of simvastatin may be more evident in regenerative and histological parameters than in early mechanical stability [44]. Despite these encouraging findings, the included studies differed substantially in design, dosage, delivery vehicle, regenerative protocol, comparator, and follow-up duration, which limits direct comparability and prevents firm conclusions regarding an optimal simvastatin protocol.

Risk of bias assessment

Randomized Controlled Trials

**Figure 2:**
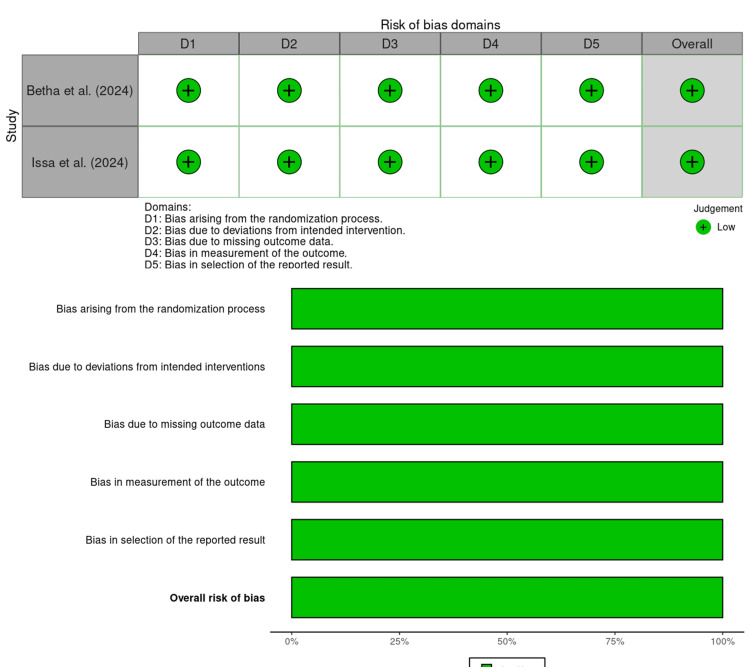
Risk of bias across the included randomized controlled trials using the Cochrane RoB 2 tool [45,48]

The two RCTs, Betha et al. (2024) [45] and Issa et al. (2024) [48], demonstrated low risk of bias across all five domains (Figure 2). This included appropriate randomization, adherence to intended interventions, low levels of missing data, acceptable outcome assessment, and transparent reporting. These studies therefore provide the strongest available human evidence within the present review.

Non-randomized Clinical Study

The non-randomized split-mouth clinical trial by El Shafei et al. (2022) [44] was evaluated using the ROBINS-I tool. While most domains, including participant selection, classification of interventions, outcome measurement, and reporting, were judged to be at low risk, bias due to confounding was considered moderate, reflecting the inherent limitations of non-randomized designs even when a split-mouth approach is used. Accordingly, the overall risk of bias was judged as moderate (Table 3). This does not invalidate the study findings, but it does require more cautious interpretation than for the randomized trials.

**Table 3:** Risk of bias in the included non-randomized clinical trial

Study	Bias due to confounding	Bias in selection of participants	Bias in classification of interventions	Bias due to deviations from intended interventions	Bias due to missing data	Bias in measurement of outcomes	Bias in selection of reported results	Overall risk of bias
El Shafei et al. (2022) [44]	Moderate	Low	Low	Low	Low	Low	Low	Moderate

Animal Preclinical Studies

The two animal studies, Mansour et al. (2014) [46] and Jun et al. (2021) [47], were appraised using the SYRCLE risk-of-bias tool (Table 4). Both studies were judged to be at low risk for baseline characteristics, incomplete outcome data, and selective reporting. However, sequence generation, allocation concealment, random housing, and blinding of caregivers were either unclear or insufficiently reported, which is a common methodological limitation in preclinical animal research. Mansour et al. [46] were assigned an overall moderate risk of bias because of multiple unclear domains, whereas Jun et al. [47] showed a lower overall risk, reflecting comparatively better methodological reporting.

**Table 4:** Risk of bias in animal studies using the SYRCLE tool

Study	Sequence generation	Baseline characteristics	Allocation concealment	Random housing	Blinding of caregivers	Random outcome assessment	Blinding of outcome assessor	Incomplete outcome data	Selective outcome reporting	Other biases	Overall risk of bias
Mansour et al. (2014) [46]	Unclear	Low	Unclear	Unclear	Unclear	Unclear	Low	Low	Low	Low	Moderate
Jun et al. (2021) [47]	Unclear	Low	Unclear	Unclear	Unclear	Low	Low	Low	Low	Low	Low

Discussion

The findings of the present systematic review indicate that simvastatin offers a potential biological advantage when incorporated into implant-associated regenerative procedures, including guided bone regeneration and related peri-implant augmentation protocols performed during immediate or simultaneous implant placement. Across the included human and animal studies, simvastatin generally demonstrated the ability to enhance osteogenesis, maintain crestal bone levels, and support more favorable peri-implant tissue outcomes [44-48]. These effects can be attributed to simvastatin’s well-documented influence on bone metabolism, including its upregulation of bone morphogenetic protein-2, stimulation of osteoblastic differentiation, suppression of osteoclast-mediated resorption, and its anti-inflammatory and angiogenic properties [29-35]. The convergence of these biological pathways supports the rationale for local simvastatin delivery as a biologically active regenerative adjunct, although the currently available evidence remains limited in quantity and heterogeneity.

The included studies represented a methodologically diverse body of evidence, encompassing randomized controlled trials, a non-randomized split-mouth clinical trial, and animal experiments in dogs and rabbits [44-48]. This diversity allowed assessment of simvastatin’s effects from clinical and radiographic outcomes in humans to histological and osseointegration-related changes in preclinical models. Human randomized clinical trials provided clinically relevant evidence under treatment conditions that resemble routine implant practice, whereas animal studies offered mechanistic insight into bone healing and bone-to-implant contact that cannot be ethically obtained from human specimens. Despite differences in study design, simvastatin delivery method, and comparator protocols, the overall direction of findings was generally favorable, which strengthens the biological plausibility of its role in peri-implant bone regeneration.

The settings and participant characteristics of the clinical studies provide some support for translational relevance, but they also highlight important limits to generalizability. The included human studies were conducted in Egypt and India, while the animal evidence originated from Egypt and South Korea [44-48]. Participants were medically healthy adults, and studies generally excluded smokers and individuals with systemic conditions known to compromise healing. While this strengthens internal validity, it also means that the findings cannot yet be confidently extended to medically compromised patients, older individuals, or more complex implant cases. These are precisely the patient groups in whom an effective biologically active adjunct might be most valuable, and they should therefore be prioritized in future clinical trials.

Variations in simvastatin delivery methods were also notable across the included studies. Simvastatin was incorporated into gels, platelet-rich fibrin matrices, graft materials, and implant surface coatings [44-48]. These differing approaches demonstrate versatility but also make it difficult to identify the most effective formulation, carrier, or concentration. Nevertheless, the available data suggest that localized simvastatin can positively influence several regenerative parameters, including crestal bone preservation, bone width gain, bone mineral density, bone-to-implant contact, and vascularized new bone formation. At the same time, the present review does not support the conclusion that all delivery systems are equally effective, since direct head-to-head comparisons are lacking and the protocols differed substantially between studies.

The outcome pattern across the included studies further supports simvastatin’s beneficial role, particularly for hard tissue regeneration. Radiographic and clinical assessments showed reduced peri-implant bone loss, improved bone width, and higher bone density in simvastatin-treated groups [44,45,48]. Histological analyses in animal studies revealed more organized trabecular bone, increased osteocyte numbers, and enhanced vascularity compared with control sites [46,47]. Some clinical studies also demonstrated favorable soft tissue responses, including reduced probing depth, lower bleeding scores, and improved esthetic parameters [45,48]. However, implant stability measured by ISQ did not improve consistently [44], suggesting that simvastatin’s main contribution may lie more in biological tissue regeneration than in immediate mechanical stabilization. This distinction is clinically important because enhanced bone healing does not necessarily translate into measurable short-term changes in stability values.

The findings of the present review are broadly in line with previous literature describing simvastatin as a promising osteopromotive adjunct in oral and maxillofacial regeneration. Prior reviews have discussed the role of simvastatin in bone tissue metabolism, carrier-based local delivery systems, and regenerative applications in dentistry more broadly [37-39]. However, most of that literature has addressed simvastatin in generalized bone regeneration, periodontal defects, or experimental models rather than specifically in implant-associated regenerative procedures. The present review, therefore, adds a more focused perspective by examining studies directly related to immediate implant placement, simultaneous implant placement, or peri-implant defect regeneration. Even so, the current evidence remains too sparse to conclude that simvastatin should be routinely incorporated into all GBR protocols, and its clinical value should still be regarded as promising rather than established.

Several limitations of the present review should be acknowledged. First, only five studies met the inclusion criteria, which substantially limits the strength of evidence. Second, the included studies had small sample sizes, mixed clinical and preclinical designs, variable simvastatin concentrations and carriers, and follow-up periods ranging from only two weeks to 12 months [44-48]. Third, the regenerative protocols were not uniform; some studies used grafts and membranes, whereas others evaluated PRF, direct socket placement, or implant coating strategies. This clinical and methodological heterogeneity reduced comparability and prevented meta-analysis. In addition, although no language restriction was applied at the search stage, the available evidence base remained narrow and may still not fully represent all relevant regenerative contexts. These limitations mean that the current conclusions should be interpreted cautiously.

From a clinical standpoint, simvastatin appears to be a low-cost and biologically plausible adjunct that may improve peri-implant regenerative outcomes when used locally in appropriate carriers. However, the present evidence does not yet identify an optimal dose, carrier system, membrane combination, or application protocol for routine practice. Future randomized controlled trials should therefore aim to standardize simvastatin concentration, delivery vehicle, grafting protocol, membrane use, follow-up intervals, and core outcome measures such as marginal bone level, bone width gain, bone density, esthetic outcomes, and implant stability. Studies with longer follow-up and larger, more diverse patient populations will be especially important to determine whether the promising short- to mid-term regenerative benefits of simvastatin translate into durable clinical advantages.

Overall, the current evidence suggests that simvastatin may serve as a useful adjunct to implant-related regenerative procedures by enhancing bone regeneration, improving bone quality, and supporting favorable peri-implant healing. Nevertheless, because the evidence base is limited and heterogeneous, simvastatin should presently be regarded as an emerging adjunctive option rather than a definitively established component of routine GBR practice. Further well-designed clinical trials are required before firm clinical recommendations can be made.

## Conclusions

The findings of this systematic review indicate that simvastatin, when used as a localized adjunct to guided bone regeneration or related implant-associated regenerative procedures, may improve hard and soft tissue outcomes by promoting osteogenesis, improving bone density and width, and supporting favorable peri-implant healing. Across the included studies, simvastatin demonstrated generally favorable biological effects, including increased bone-to-implant contact and reduced crestal bone loss, without reported major adverse effects. Although its impact on implant stability was not uniformly significant, the overall findings suggest that simvastatin has potential as a useful supplement to conventional regenerative implant protocols. However, the current evidence base is limited by the small number of studies, methodological heterogeneity, and variation in simvastatin concentration, carrier systems, and follow-up periods. Further well-designed clinical trials with larger sample sizes, standardized concentrations, uniform delivery systems and outcome measures, and longer follow-up periods are required to define optimal clinical protocols and confirm the long-term benefits of incorporating simvastatin into regenerative implant therapy.

## Appendices

Table 5 presents the complete search strategies used in the present study.

**Table 5:** Database-wise electronic search strategies used in the present systematic review

Database/source	Coverage period	Search strategy
PubMed/MEDLINE	Inception to April 30, 2025	(("Simvastatin"[Mesh] OR simvastatin[tiab] OR statin[tiab]) AND ("Dental Implants"[Mesh] OR "Immediate Dental Implant Loading"[Mesh] OR "dental implant"[tiab] OR "immediate implant*"[tiab] OR "simultaneous implant placement"[tiab] OR "implant placement"[tiab] OR "peri-implant"[tiab]) AND ("Guided Tissue Regeneration, Periodontal"[Mesh] OR "Bone Regeneration"[Mesh] OR "guided bone regeneration"[tiab] OR GBR[tiab] OR "bone regeneration"[tiab] OR "ridge preservation"[tiab] OR "ridge augmentation"[tiab] OR "ridge splitting"[tiab] OR "bone graft*"[tiab] OR osteogenesis[tiab] OR osseointegration[tiab]))**
Embase	Inception to April 30, 2025	('simvastatin'/exp OR simvastatin:ti,ab OR statin:ti,ab) AND ('dental implant'/exp OR 'immediate implant loading'/exp OR 'dental implant':ti,ab OR 'immediate implant*':ti,ab OR 'simultaneous implant placement':ti,ab OR 'implant placement':ti,ab OR 'peri-implant':ti,ab) AND ('bone regeneration'/exp OR 'guided bone regeneration':ti,ab OR gbr:ti,ab OR 'bone regeneration':ti,ab OR 'ridge preservation':ti,ab OR 'ridge augmentation':ti,ab OR 'ridge splitting':ti,ab OR 'bone graft*':ti,ab OR osteogenesis:ti,ab OR osseointegration:ti,ab)**
Scopus	Inception to April 30, 2025	TITLE-ABS-KEY ((simvastatin OR statin) AND ("dental implant" OR "immediate implant*" OR "simultaneous implant placement" OR "implant placement" OR "peri-implant") AND ("guided bone regeneration" OR GBR OR "bone regeneration" OR "ridge preservation" OR "ridge augmentation" OR "ridge splitting" OR "bone graft*" OR osteogenesis OR osseointegration))**
Web of Science Core Collection	Inception to April 30, 2025	TS=((simvastatin OR statin) AND ("dental implant" OR "immediate implant*" OR "simultaneous implant placement" OR "implant placement" OR "peri-implant") AND ("guided bone regeneration" OR GBR OR "bone regeneration" OR "ridge preservation" OR "ridge augmentation" OR "ridge splitting" OR "bone graft*" OR osteogenesis OR osseointegration))**
Cochrane CENTRAL	Inception to April 30, 2025	(simvastatin OR statin):ti,ab,kw AND ("dental implant" OR "immediate implant*" OR "simultaneous implant placement" OR "implant placement" OR "peri-implant"):ti,ab,kw AND ("guided bone regeneration" OR GBR OR "bone regeneration" OR "ridge preservation" OR "ridge augmentation" OR "ridge splitting" OR "bone graft*" OR osteogenesis OR osseointegration):ti,ab,kw**
Google Scholar	Up to April 30, 2025	Searches were performed using combinations of the following terms: "simvastatin guided bone regeneration dental implants", "simvastatin immediate implant placement", "simvastatin bone regeneration implant", "simvastatin ridge splitting implant", and "simvastatin peri-implant bone regeneration". The first 200 results sorted by relevance were screened.
ClinicalTrials.gov	Up to April 30, 2025	Search terms included the following: simvastatin AND dental implant, simvastatin AND guided bone regeneration, simvastatin AND bone regeneration, and simvastatin AND implant placement.
Manual search	Up to April 30, 2025	The reference lists of all included studies and relevant review articles were screened manually to identify additional eligible studies not retrieved through the electronic database search.
